# Juvenile hormone biosynthesis in adult *Blattella germanica* requires nuclear receptors Seven-up and FTZ-F1

**DOI:** 10.1038/srep40234

**Published:** 2017-01-11

**Authors:** Ferran Borras-Castells, Claudia Nieva, José L. Maestro, Oscar Maestro, Xavier Belles, David Martín

**Affiliations:** 1 Institute of Evolutionary Biology (CSIC-Universitat Pompeu Fabra) Passeig Marítim de la Barceloneta 37-49, 08003 Barcelona, Spain

## Abstract

In insects, the transition from juvenile development to the adult stage is controlled by juvenile hormone (JH) synthesized from the *corpora allata* (CA) glands. Whereas a JH-free period during the last juvenile instar triggers metamorphosis and the end of the growth period, the reappearance of this hormone after the imaginal molt marks the onset of reproductive adulthood. Despite the importance of such transition, the regulatory mechanism that controls it remains mostly unknown. Here, using the hemimetabolous insect *Blattella germanica*, we show that nuclear hormone receptors Seven-up-B (BgSvp-B) and Fushi tarazu-factor 1 (BgFTZ-F1) have essential roles in the tissue- and stage-specific activation of adult CA JH-biosynthetic activity. Both factors are highly expressed in adult CA cells. Moreover, RNAi-knockdown of either *BgSvp-B* or *BgFTZ-F1* results in adult animals with a complete block in two critical JH-dependent reproductive processes, vitellogenesis and oogenesis. We show that this reproductive blockage is the result of a dramatic impairment of JH biosynthesis, due to the CA-specific reduction in the expression of two key JH biosynthetic enzymes, *3-hydroxy-3-methylglutaryl coenzyme A synthase-1 (BgHMG-S1*) and *HMG-reductase (BgHMG-R*). Our findings provide insights into the regulatory mechanisms underlying the specific changes in the CA gland necessary for the proper transition to adulthood.

Postembryonic development in winged insects is divided into three stages: (i) a juvenile phase where growth takes place, (ii) the metamorphic transformation during the last juvenile instar, and (iii) the reproductive adulthood. Juvenile hormone (JH), a sesquiterpenoid hormone produced and released by the *corpora allata* (CA) glands, controls critical events that occur during these three stages and also the transitions between them^1^,^2^,^3^. Thus, the presence of JH during pre-ultimate juvenile stages prevents the premature differentiation of adult features, while its disappearance upon entering into the last immature stage allows metamorphosis to occur^4^. Finally, the reappearance of high amounts of JH after the imaginal molt marks the onset of the adult period. During this stage, JH controls critical aspects of the reproductive process, such as yolk protein synthesis and oocyte maturation^5^,^6^,^7^. Despite the importance of the precise fluctuations of JH levels during insect development, the molecular mechanisms underlying the regulation of JH biosynthesis remain poorly understood.

JH biosynthesis involves a series of enzymatic reactions that are conventionally organized into two modules. In the first one, known as the mevalonate pathway, acetyl-CoA is transformed into farnesyl pyrophosphate, while the reactions in the second module are responsible for the conversion of farnesyl pyrophosphate into JH^8^. The genes encoding the enzymes involved in both modules are highly enriched, or even exclusively expressed, in the CA cells^9^. In the lepidopteran *Bombyx mori*, the dipteran *Aedes aegypti* and the dictyopteran *Diploptera punctata*, the expression patterns of JH biosynthesis enzymes in the CA cells correlate well to the JH synthetic activity of the gland, suggesting that the fluctuations in JH synthesis are mainly controlled at the level of the expression of these enzymes^9^,^10^,^11^,^12^,^13^. Unfortunately, very few factors have been connected to the transcriptional control of JH synthesis to date. In the dipteran *Drosophila melanogaster*, TGFbeta signaling stimulates JH production by up-regulating the transcription of the *JH acid methyltransferase (JHAMT*) gene through the transcription factor Mothers against Dpp (Mad)^14^. In the coleopteran *Tribolium castaneum*, the POU factor Ventral veins lacking/Drifter (vvl) activates JH synthesis during larval stages by activating *JHAMT3* expression^15^. In addition to these transcription factors, several peptide hormones (allatotropins, allatostatins and short neuropeptide F) and neurotransmitters (the biogenic amines octopamine, dopamine and glutamate) are also involved in the regulation of CA activity^16^. However, although these factors regulate JH synthesis to some extent, the transcriptional regulatory network that control the expression of key JH biosynthetic genes during the different stages of development remains to be defined.

Here, we use the hemimetabolous insect *Blattella germanica* to elucidate the molecular mechanisms underlying the critical reactivation of JH production that marks the onset of the reproductive adulthood. This cockroach provides an ideal opportunity to address JH biosynthesis regulation as patterns and functions of JH III, the only JH form detected in this species^17^, have been intensively studied during the nymphal and adult stages^18^,^19^. The successive gonadotrophic cycles that conform the adult period of *B. germanica* females are characterized by a very high rate of JH synthesis, which induces the massive production of yolk precursor proteins by the fat body, as well as the incorporation of these proteins into developing oocytes^7^,^20^,^21^,^22^,^23^. Concomitantly with the strong increase in the rate of JH biosynthesis after the imaginal molt, the CA cells of *B. germanica* undergo a specific physiological change during this stage, switching from the proliferative phase characteristic of the juvenile development to an adult-specific non-proliferative state^24^,^25^.

In the present study, by using a detailed RNA interference (RNAi) analysis, we uncover the specific regulatory role of two transcription factors that controls JH synthesis and cell proliferation in the CA of adult *B. germanica*. Our findings reveal that nuclear hormone receptors Seven-up-B (BgSvp-B) and Fushi tarazu-factor 1 (BgFTZ-F1) provide tissue- and stage-specificity to the expression of two key JH biosynthetic enzyme genes, *3-hydroxy-3-methylglutaryl coenzyme A synthase-1 (BgHMG-S1*) and *3-hydroxy-3-methylglutaryl coenzyme A reductase (BgHMG-R*), in the CA cells to control the massive production of JH during *B. germanica* adulthood. We also provide evidence that BgSvp-B is required to maintain the proliferative capacity of CA cells during nymphal development. Thus, our work identifies the transcription factors that generate the physiological and functional changes in the CA gland necessary for the proper transition to the adult stage and the reproductive adulthood.

## Results

### Cloning and developmental expression of BgSvp isoforms

In search of transcription factors that might regulate the activity of CA cells in *B. germanica*, nuclear receptor Svp emerged as a good candidate as it has been found to be one of the few factors strongly and specifically expressed in the *corpus allatum* of *D. melanogaster* embryos^26^,^27^. After a PCR step using degenerated primers designed in conserved regions of insect *Svp* sequences and subsequent 5′- and 3′-RACE-PCR methodologies, we obtained two cDNAs from *B. germanica*. The sequences, differing only in the C-terminal region, corresponding to the ligand binding domain (LBD), were named *BgSvp-A* (accession number: KT423097) and *BgSvp-B* (accession number: KT423098) based on the similarity of their DNA-binding domain (DBD) and LBD with other insect *Svp* (Supplementary Fig. S1). BgSvp-B has all the domains of the nuclear receptor family and shares more than 90% identity in the DBD and LBD with other insect Svp and with its vertebrate homolog, chicken ovalbumin upstream promoter transcription factor 1 (COUP-TF1). BgSvp-A is identical to BgSvp-B except for the last 90 amino acids of the LBD. Instead, BgSvp-A presents a short 21 amino acid stretch with no homology to other Svp proteins or known nuclear factors (Supplementary Fig. S1).

**Figure 1:**
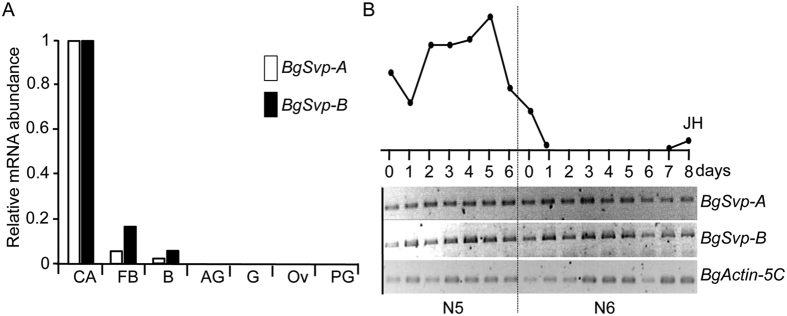
BgSvp isoforms are highly expressed in the CA of *B. germanica*. (**A**) Expression levels of *BgSvp-A*, and *BgSvp-B* relative to *BgActin5C* in different tissues of 8-old-day *B. germanica* last instar nymph females, measured by qRT-PCR. *corpora allata* (CA), ovary (Ov), fat body (FB), brain (**B**), accesory glands (AG), prothoracic gland (PG), and gut (G). Fold changes are relative to the expression of each gene in the CA, arbitrarily set to 1. (**B**) Expression patterns of *BgSvp-A and BgSvp-B* mRNAs in the CA during the last two nymphal instars of *B. germanica*, analyzed by semi-quantitative RT-PCR. *BgActin5C* mRNA levels were used as a reference. The blot is representative of three replicates. Circulating JH levels (upper part) are redrawn from ref. 19.

As a first step towards the characterization of *BgSvp* functions, the mRNA levels of *BgSvp-A* and *BgSvp-B* were analyzed in different tissues of *B. germanica*. Consistent with a possible role in the CA, both *BgSvp* isoforms were strongly expressed in these glands. They were also detected in the fat body and brain although to a lesser extent, while no expression was detected in the other tissues analyzed (Fig. 1A). Given the tissue specificity, *BgSvp* isoforms expression was investigated further in the CA and found that *BgSvp-A* and *BgSvp-B* mRNAs persisted in these glands without major variations during *B. germanica* development (Fig. 1B).

### BgSvp nuclear receptors play a central role in the adult stage of *B. germanica*

To examine the role of BgSvp isoforms, we used systemic RNA interference (RNAi) to knockdown *BgSvp* during nymphal development. First, we lowered the expression of both receptors simultaneously by injecting a dsRNA encompassing the common LBD region (*dsBgSvp-1*; Supplementary Fig. S2A). *dsBgSvp-1* was injected in newly emerged last instar female nymphs (herein called *BgSvpi* animals), and mRNA levels of *BgSvp-A* and *BgSvp-B* in the CA were determined 7 days later. Specimens injected with *dsMock* were used as negative controls (*Control* animals). The mRNA levels of both receptors decreased substantially in *BgSvpi* insects compared to *Control* animals (75% for *BgSvp-A*, and 81% for *BgSvp-B*) (Supplementary Fig. S2B,C).

**Figure 2:**
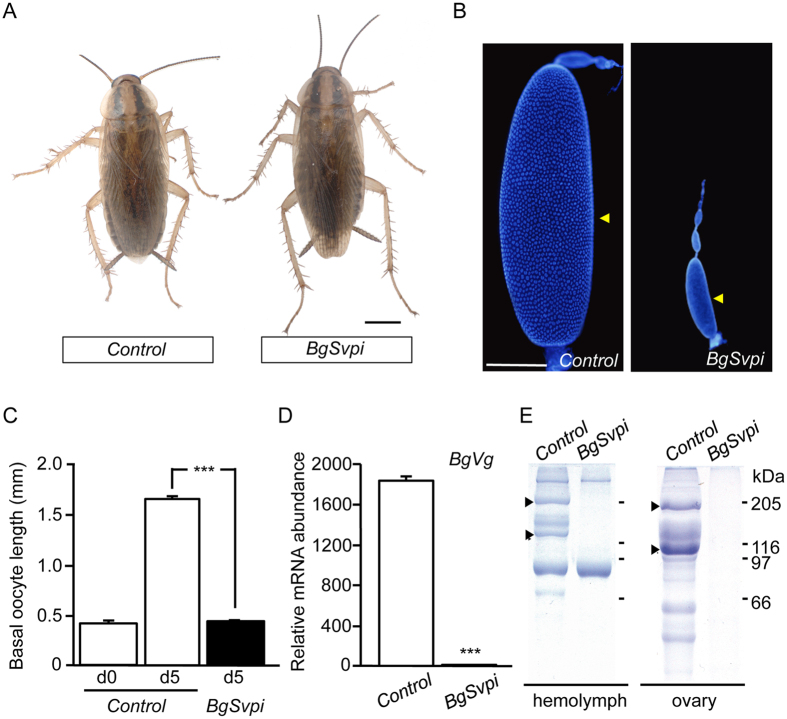
Loss of BgSvp impairs reproduction in *B. germanica* adult females. (**A**) Effect of RNAi of *BgSvp*. Newly emerged sixth instar female nymphs were injected with 3 μg of *dsMock (Control*) or *dsBgSvp-1 (BgSvpi*). Dorsal views of *Control* and *BgSvpi* animals showing normal winged adult appearance. (**B**) DAPI-stained ovarioles from 5-day-old *Control* and *BgSvpi* adult females. Arrowheads indicate basal oocytes. (**C**) Basal oocyte length of *Control* and *BgSvpi* adult females of the indicated age. Results are expressed as the mean ± S.E. (n = 25–31). (**D**) *BgVg* mRNA levels in the fat body of 5-day-old *Control* and *BgSvpi* females, relative to *BgActin5C* mRNA levels, measured by qRT-PCR. Error bars represent SEM (n = 4). Asterisks in (**C** and **D**) indicate differences statistically significant as follows: ***p ≤ 0.0001 (Student’s *t* test). (**E**) SDS–PAGE of hemolymph (left) and ovaries (right) from 5-day-old *Control* and *BgSvp-1i* adult females. Arrowheads indicate BgVg subunits. Scale bars: 1 mm in (**A**); 200 μm in (**B**).

All *BgSvpi* nymphs (*n* = 124) molted properly into adults and had normal appearance, as *Control* nymphs (*n* = 50) (Fig. 2A). This allowed us to analyze the role of BgSvp during the adult period. The first gonadotrophic cycle of *B. germanica* starts just after the imaginal molt and lasts 8 days, during which JH induces the synthesis of yolk protein precursors in the fat body. The yolk proteins are then released to the hemolymph and subsequently incorporated to the growing basal oocytes in a JH-dependent manner. As a result of the massive incorporation of yolk proteins, the basal oocytes show an exponential growth during each gonadotrophic cycle. Then, at the end of the cycle, eggs are oviposited into an egg-case or ootheca that is transported by the female during 18 days until egg hatching^7^,^20^,^21^,^22^. Notably, whereas all *Control* adult females oviposited eight days after the imaginal molt, none of the *BgSvpi* animals oviposited nor produced any sign of ootheca formation. A detailed examination of the *BgSvpi* females 5 days after the imaginal molt revealed a complete impairment of egg development (Fig. 2B,C). Given that oocyte growth depends on the accumulation of large amounts of the yolk protein vitellogenin (BgVg), we next measured the expression of *BgVg* gene in the fat body of *BgSvpi* adults. Compared to *Control* adults, *BgVg* mRNA levels in *BgSvpi* animals were dramatically reduced (Fig. 2D) and, consequently, BgVg protein was neither detected in the hemolymph nor within the oocytes (Fig. 2E). Altogether, our results show that *BgSvp* isoforms play a central role in the reproduction of *B. germanica* adult females.

In order to characterize whether the requirement of BgSvp was exclusive of the adult stage or it was also necessary during the pre-ultimate nymphal stages, *dsBgSvp-1* was injected into the abdomen of freshly ecdysed fourth (antepenultimate) or fifth (penultimate) instar nymphs. Under these conditions, 100% of *BgSvpi* nymphs (*n* = 30) developed normally and underwent two or three successive molts, depending at which instar they had been injected, until reaching adulthood properly, indicating that *BgSvp* isoforms are not involved in vital processes during nymphal development. Eventually, the *BgSvpi* adults showed the same reproductive impairment observed in *BgSvpi* animals injected in the last nymphal instar.

### BgSvp-B is the isoform required for reproduction in adult *B. germanica*

**Figure 3:**
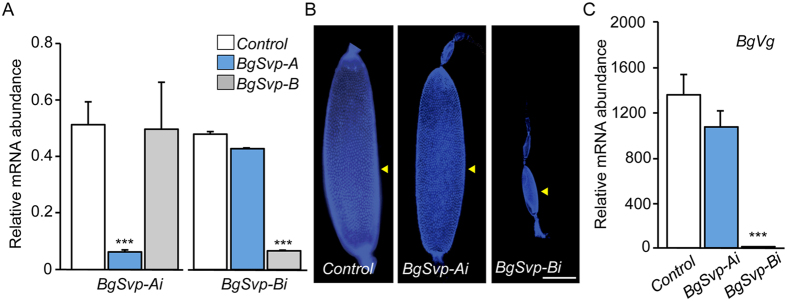
*BgSvp-B* is the isoform necessary for adult reproduction in *B. germanica*. (**A**–**C**) Effect of BgSvp isoform-specific RNAi. Newly emerged sixth instar female nymphs were injected with 3 μg of *dsMock (Control*), *dsBgSvp-A (BgSvp-Ai*) or *dsBgSvp-B (BgSvp-Bi*). (**A**) mRNA levels of *BgSvp-A* and *BgSvp-B*, relative to *BgActin5C* levels, in the CA of 6–day-old N6 specimens, measured by qRT-PCR. (**B**) DAPI-stained ovarioles from 5-day-old *Control, BgSvp-Ai* and *BgSvp-Bi* adult females. Arrowheads indicate basal oocytes. (**C**) *BgVg* mRNA levels in the fat body of 5-day-old *Control, BgSvp-Ai*, and *BgSvp-Bi* females, relative to *BgActin5C* mRNA levels, measured by qRT-PCR. Error bars in (**A** and **C**) represent SEM (n = 5); asterisks in (**A** and **C**) indicate differences statistically significant with respect to *Controls* (***p ≤ 0.0001 (Student’s *t* test)). Scale bar in (**B**): 200 μm.

To assess the role of each *BgSvp* isoform on the *BgSvpi* phenotype, dsRNAs targeting each isoform (*dsBgSvp-A* and *dsBgSvp-B*; Fig. S2A) were injected separately into newly emerged last instar nymphs (*BgSvp-Ai* and *BgSvp-Bi* animals, respectively). The treatment resulted in a remarkably decrease in the corresponding transcript without affecting the expression of the other isoform (Fig. 3A). As in *BgSvpi* animals, all the isoform-specific knockdown nymphs developed normally and molted to the adult stage without any observable defect (*n* = 30 for *BgSvp-Ai*; *n* = 40 for *BgSvp-Bi*). After completing the first gonadotrophic cycle, *Control* and *BgSvp-Ai* females successfully oviposited while *BgSvp-Bi* females were unable to lay eggs as they showed a drastic impairment in egg development and reduced *BgVg* expression in the fat body (Fig. 3B,C). Altogether, these results show that BgSvp-B is the responsible for the reproductive impairment observed in the *BgSvpi* animals.

### BgSvp-B controls JH production by regulating the expression of JH biosynthetic enzyme genes

**Figure 4:**
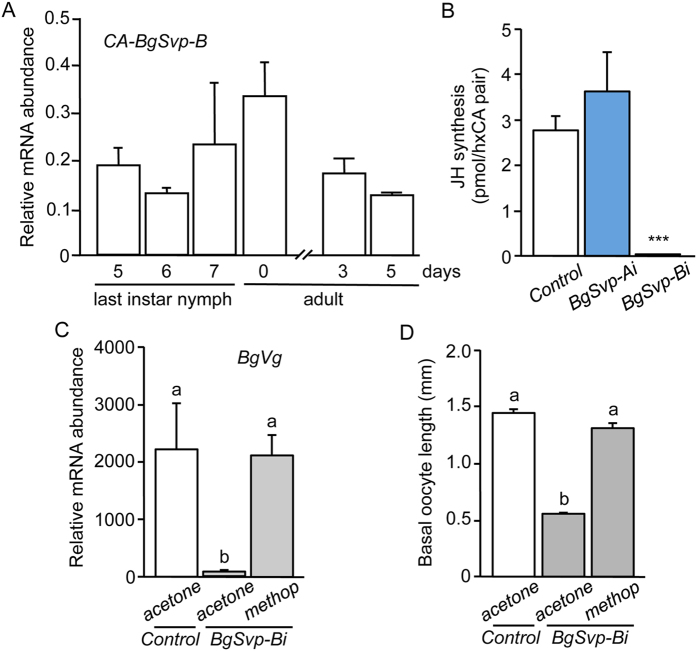
BgSvp-B controls JH production in *B. germanica* adult females. (**A**) Expression patterns of *BgSvp-B*, relative to *BgActin5C* levels, in the CA of *B. germanica* females during the last nymphal instar and the adult stage, measured by qRT-PCR. (**B**–**D**) Newly emerged sixth instar female nymphs were injected with 3 μg of *dsBgSvp-A (BgSvp-Ai*), *dsBgSvp-B (BgSvp-Bi*) or with *dsMock (Control*). (**B**) Rates of JH synthesis by CA incubated *in vitro* from 5-day-old *Control, BgSvp-Ai* and *BgSvp-Bi* adult females. (**C**) Expression levels of *BgVg*, relative to *BgActin5C* levels, in the fat body of 5-day-old *BgSvp-Bi* adult females treated with acetone or methoprene, compared to acetone-treated *Control* adults, measured by qRT-PCR. (**D**) Basal oocyte length of 5-day-old *BgSvp-Bi* adult females treated with acetone or methoprene, compared to acetone-treated *Control* adults. Error bars represent SEM (n = 3–10). Asterisks in (**B**) indicate differences statistically significant with respect to *Controls* (***p ≤ 0.0001; Student’s *t* test). Different letters in (**C** and **D**) represent groups with significant differences according to ANOVA test (Tukey, *p* ≤ 0.005).

Given the high expression of *BgSvp-B* in the CA and that the defects observed in *BgSvp-Bi* adults are suggestive of a JH deficiency, we next studied whether *BgSvp-B* is crucial for controlling the biosynthesis and/or the sensitivity to JH during the adult stage. To this aim, we first confirmed that *BgSvp-B* was expressed in the CA cells of adult *B. germanica* females (Fig. 4A). Then, we measured JH production by the CA from 5-day-old *BgSvp-Bi* adults and found that JH synthesis was drastically reduced compared to *BgSvp-Ai* and *Control* animals (Fig. 4B). To analyze whether JH sensitivity was also compromised in *BgSvp-Bi* adults, we topically applied methoprene, a potent JH mimic, in newly ecdysed *BgSvp-Bi* adults. This treatment restored normal levels of *BgVg* expression in the fat body and the growth of the primary follicles (Fig. 4C,D), indicating that the adult phenotype of *BgSvp-Bi* animals is due to impaired JH synthesis but not to the transduction of the JH signal. Taken together, these results show that *BgSvp-B* plays a critical role in the reproduction of *B. germanica* by controlling JH biosynthesis in the CA during the adult stage.

**Figure 5:**
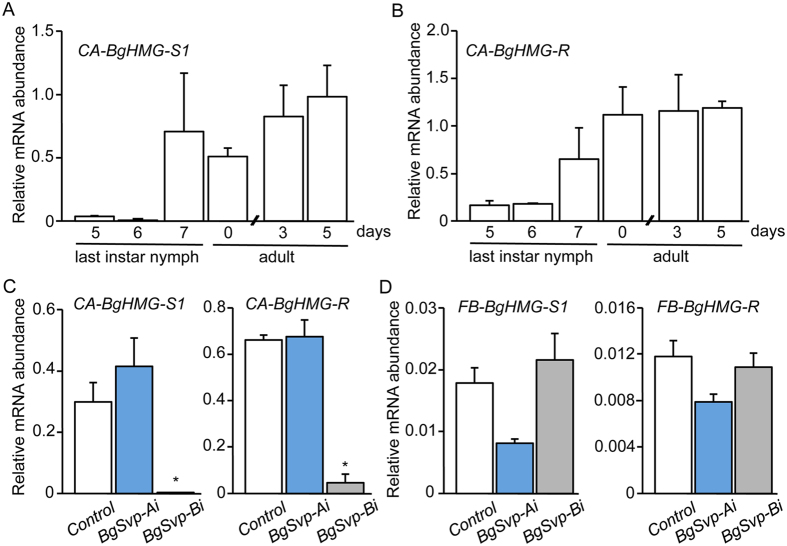
BgSvp-B controls the expression of JH biosynthetic enzyme genes in the CA of *B. germanica* adult females. (**A** and **B**) Expression patterns of *BgHMG-S1* (**A**) and *BgHMG-R* (**B**), relative to *BgActin5C* levels, in the CA of *B. germanica* females during the last nymphal instar and the adult stage, measured by qRT-PCR. (**C**) *BgHMG-S1* and *BgHMG-R* mRNA levels in the CA of 5-day-old *Control, BgSvp-Ai* and *BgSvp-Bi* females, relative to *BgActin5C* mRNA levels, measured by qRT-PCR. (**D**) *BgHMG-S1* and *BgHMG-R* mRNA levels in the fat body of *Control, BgSvp-Ai* and *BgSvp-Bi* females, relative to *BgActin5C* mRNA levels. Error bars represent SEM (n = 6). Asterisks in (**C**) indicate differences statistically significant with respect to *Controls* (*p ≤ 0.0001; Student’s *t* test).

**Figure 6:**
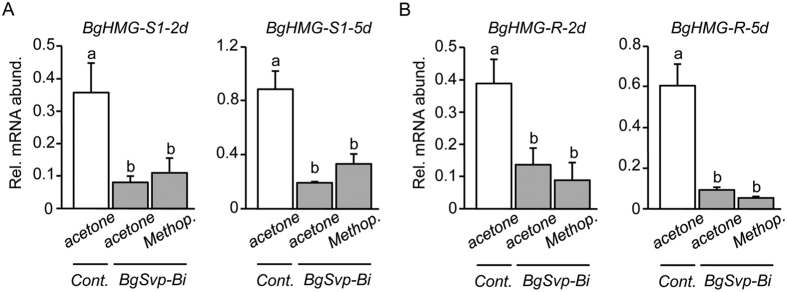
*BgHMG-S1* and *BgHMG-R* reduction in CA of *BgSvp-Bi* animals is not due to the absence of JH. (**A** and **B**) Expression levels of *BgHMG-S1* (**A**), and *BgHMG-R* (**B**), relative to *BgActin5C* levels, in the CA of 2 and 5-day-old *BgSvp-Bi* adult females treated with acetone or methoprene, compared to acetone-treated *Control* adults. Error bars represent SEM (n = 3–5). Different letters represent groups with significant differences according to ANOVA test (Tukey, *p* ≤ 0.05).

To address how BgSvp-B controls JH biosynthesis, we next asked whether it regulates the expression of genes encoding crucial enzymes of the pathway. In *B. germanica*, BgHMG-S1 and BgHMG-R have been identified as key enzymes in the mevalonate pathway leading to JH synthesis^28^,^29^,^30^. *B. germanica* has a second HMG synthase gene, *BgHMG-S2*, although its expression and regulation is identical to *BgHMG-S1*^30^,^31^. Paralleling the rate of JH synthesis, mRNAs of *BgHMG-S1* and *BgHMG-R* are significantly upregulated in the CA during the nymphal-to-adult transition and are maintained high during the first gonadotrophic cycle (Fig. 5A,B). Thus, we next examined whether *BgSvp-B* depletion affects the expression of *BgHMG-S1* and *BgHMG-R* during the adult stage. Remarkably, *BgHMG-S1* and *BgHMG-R* mRNA levels in the CA of *BgSvp-Bi* adults were severely reduced compared to *BgSvp-Ai* and *Control* adults (Fig. 5C). In contrast, mRNA levels of *BgHMG-S1* and *BgHMG-R* in the fat body of *BgSvp-Bi* adults did not differ from those in *Control* and *BgSvp-Ai* animals (Fig. 5D), suggesting that the regulatory effect of BgSvp-B is CA-specific. To confirm that the reduced levels of *BgHMG-S1* and *BgHMG-R* in the CA were due to the absence of *BgSvp-B* and not because of the low titer of JH, we applied methoprene to newly ecdysed *BgSvp-Bi* adults and the mRNA levels of both enzymes were analyzed 2 and 5 days later. As Fig. 6 shows, methoprene was not able to induce *BgHMG-S1* and *BgHMG-R* expression when *BgSvp-B* levels are reduced. Overall, our data show that BgSvp-B is necessary in adult CA cells for the specific up-regulation of, at least, two important JH biosynthesis enzyme genes, *BgHMG-S1* and *BgHMG-R*, in order to support the high rate of JH synthesis associated to adulthood in *B. germanica.*

### BgSvp-B controls the proliferation of CA cells during nymphal development

**Figure 7:**
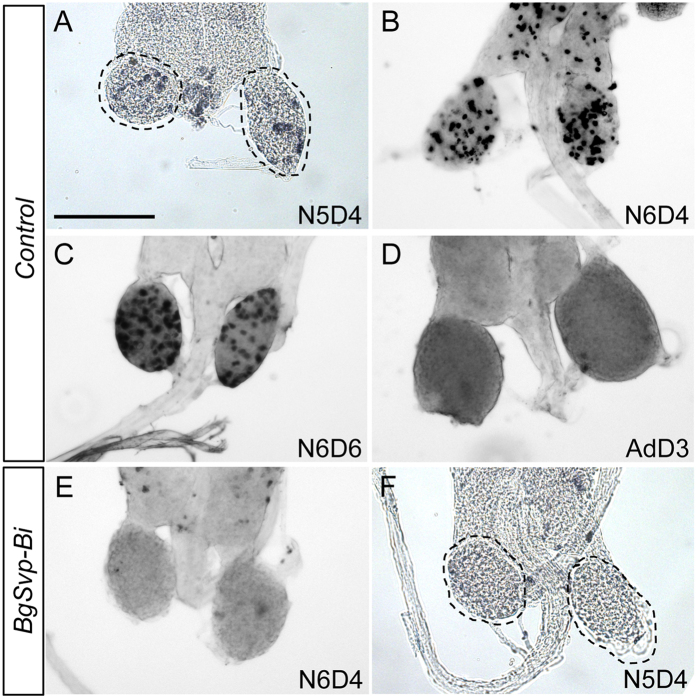
BgSvp-B promotes proliferation of CA cells during nymphal development in *B. germanica*. *Control* and *BgSvp-Bi* animals were pulsed with BrdU and the CA were dissected and stained to reveal BrdU incorporation. CA from penultimate (N5) (**A**), and last (N6) nymphal stages at the indicated ages (**B** and **C**) show abundant BrdU-labeled cells, while CA from adult females (**D**) show no BrdU incorporation. (**E** and **F**) CA from N6 and N5 *BgSvp-Bi* nymphs do not show any BrdU-labeled cells. Scale bar in (**A**): 200 μm.

The CA cells of *B. germanica* proliferate during the successive nymphal stages and switch to an adult-specific non-proliferative state immediately after the imaginal molt^24^,^25^ (Fig. 7A–D). Given the continuous and strong expression of *BgSvp-B* in nymphal CA cells (Fig. 1B), we wondered whether BgSvp-B is involved in the control of CA cell proliferation during nymphal development. To test this possibility, we injected BrdU into staged last instar *BgSvp-Bi* and *Control* nymphs and analyzed its incorporation into CA cells 24 h later. Notably, CA cells from *BgSvp-Bi* nymphs did not show any BrdU-labeling in contrast to *Control* animals (Fig. 7E). The same result was obtained when BrdU incorporation was measured in CA cells of *BgSvp-Bi* penultimate instar nymphs (Fig. 7F). Overall, our data show that, in addition to its critical role in the transcriptional control of JH biosynthetic enzymes during the adult stage, BgSvp-B is also necessary for maintaining the continuous proliferation of the CA cells during nymphal development.

**Figure 8:**
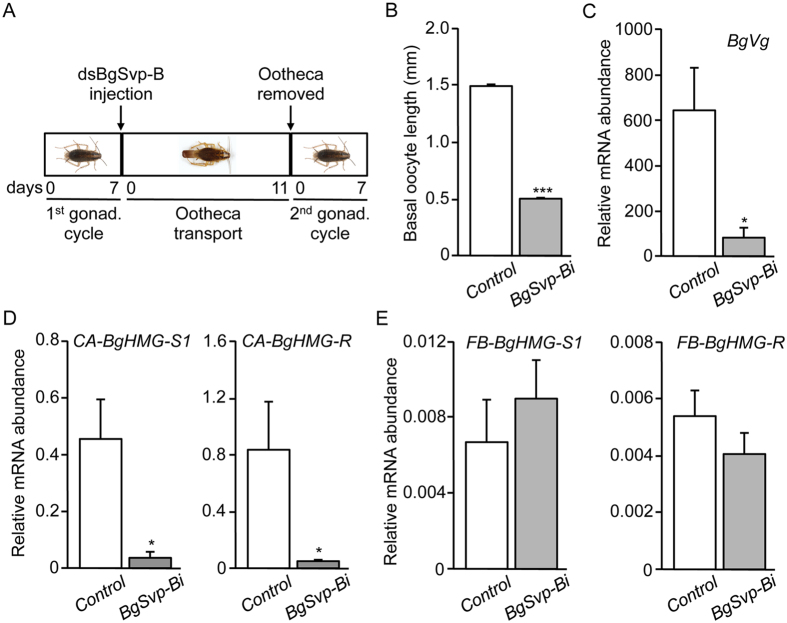
BgSvp-B controls JH production in the second gonadotrophic cycle of *B. germanica*. (**A**) Diagram of the experimental design to analyze the role of BgSvp-B in the second gonadotrophic cycle of adult *B. germanica*. (**B**) Basal oocyte length of 5-day-old *Control* and *BgSvp-1i* adult females. Results are expressed as the mean S.E. (n = 15–20). (**C**–**E**) Transcript levels of *BgVg* in the fat body (**C**), *BgHMG-S1* and *BgHMG-R* in the CA (**D**), and *BgHMG-S1* and *BgHMG-R* in the fat body (**E**) of second gonadotrophic cycle 5-day-old *Control* and *BgSvp-Bi* adults, measured by qRT-PCR. Transcript abundance values are normalized against the *BgActin5C* transcript. Error bars indicate the SEM (n = 5–10). Asterisks indicate differences statistically significant (*p ≤ 0.05; ***p ≤ 0.0005, Student’s *t*-test).

One possibility emerging from the previous result is that the impairment of JH synthesis in *BgSvp-Bi* adults might be the consequence of the reduced number of CA cells. To test this, we depleted *BgSvp-B* in the second adult gonadotrophic cycle when the final cell number in the adult CA has been already reached at the onset of the first gonadotrophic cycle. Thus, we injected *dsBgSvp-B* and *dsMock* in females during the first day of ootheca transport, and then, the ootheca was removed 11 days later to induce the onset of the second gonadotrophic cycle (Fig. 8A). Under these conditions, none of the *BgSvp-Bi* animals (n = 20) oviposited at the end of the second cycle. The detailed analysis of these animals revealed a complete block in egg development (Fig. 8B), and severely reduced levels of *BgVg* mRNA in the fat body (Fig. 8C) and *BgHMG-S1* and *BgHMG-R* in the CA (Fig. 8D). In contrast, the reduction of *BgSvp-B* levels did not affect the mRNA levels of both enzymes in the fat body (Fig. 8E). Therefore, the results were similar to those obtained with specimens treated as nymphs and analyzed in the first gonadotrophic cycle, which indicate that the impairment in JH synthesis in *BgSvp-Bi* adults is not due to a reduced CA cell number but rather to the absence of BgSvp-B.

### Nuclear receptor BgFTZ-F1 is present in CA cells during the adult period

**Figure 9:**
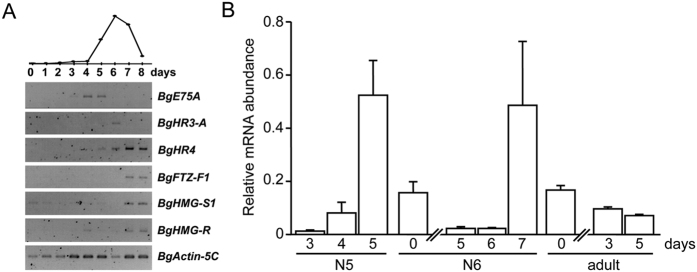
*BgFTZ-F1* is expressed in the CA of *B. germanica*. (**A**) Expression patterns of 20-hydroxyecdysone-dependent nuclear receptors during the last nymphal instar of *B. germanica*, analyzed by semi-quantitative RT-PCR. *BgActin5C* mRNA levels were used as a reference. The blot is representative of three replicates. 20-hydroxyecdysone levels (upper part) are redrawn from ref. 18. (**B**) Expression pattern of *BgFTZ-F1*, relative to *BgActin5C* levels, in the CA of *B. germanica* females during the penultimate and last nymphal instars and the adult stage, measured by qRT-PCR.

Considering the tissue-specificity and the constant developmental expression of *BgSvp-B* in the CA, it seems likely that BgSvp-B is critical for the spatial control of JH biosynthetic gene expression in the CA but not for the temporal regulation during the adult period. Searching for regulatory factors involved in the temporal control of JH synthesis, we noticed that the timing of *BgHMG-S1* and *BgHMG-R* up-regulation in the nymphal-adult transition correlates well with the decline of the 20E pulse that triggers such transition. We have previously demonstrated that a complex 20E-triggered hierarchy of nuclear receptors, converging in the strong up-regulation of *BgFTZ-F1* at the imaginal transition, regulates the imaginal molt^32^,^33^,^34^,^35^,^36^,^37^. Consistently, we found that this 20E-induced cascade of nuclear receptors, composed by *BgE75A, BgHR3-A, BgHR4*, and *BgFTZ-F1* was sequentially expressed also in last nymphal instar CA cells (Fig. 9A). Remarkably, the increase in *BgFTZ-F1* mRNA levels during the last 2 days of the instar parallel those of *BgHMG-S1* and *BgHMG-R* in this gland during the imaginal transition (Fig. 9A). Consistent with a possible role in the control of JH biosynthesis, *BgFTZ-F1* was also expressed in the CA cells during the adult stage (Fig. 9B). However, the same changes in the mRNA levels of *BgFTZ-F1* were also observed in the penultimate-to-last nymphal instar transition (Fig. 9B).

**Figure 10:**
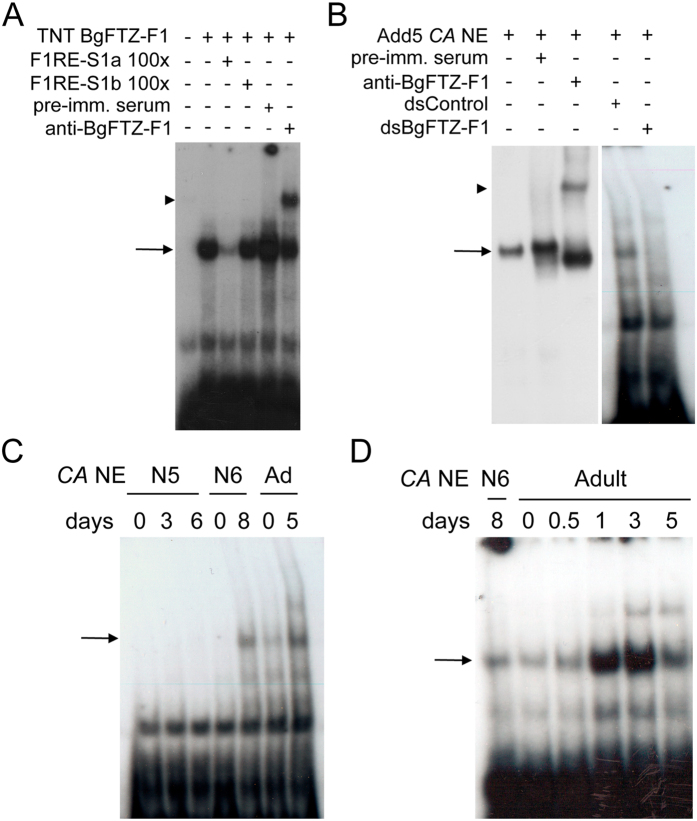
BgFTZ-F1 protein is present in the CA of *B. germanica* adult females. (**A**) EMSA analysis of the BgFTZ-F1 protein translated *in vitro*. The protein sample was incubated with ^32^P-labeled F1RE-S1a response element. The specificity of the interaction was tested by the addition of 100-fold molar excess of unlabeled F1RE-S1a and F1RE-S1b elements. The identity of the complex was tested with the addition of anti-BgFTZ-F1 antibody (anti-BgFTZ-F1) and pre-immune serum (pre-imm. serum). (**B**) Identification of BgFTZ-F1 protein in CA nuclear extracts (CA NE) of *B. germanica* 5-day-old adult females. The identity of the complex was tested with the addition of anti-BgFTZ-F1 and pre-imm serum, as well as with the analysis of CA NE from *dsBgFTZ-F1-*treated adults from the same age. (**C**) Developmental profile of BgFTZ-F1 protein present in CA NE from penultimate (N5) and last (N6) instar nymphs, as well as adult females incubated with labeled F1RE-S1a element. 20 CA equivalent was used in each binding reaction. (**D**) Developmental profile of BgFTZ-F1 protein present in CA NE from females of the first gonadotrophic cycle incubated with labeled F1RE-S1a element. Arrows in (**A**–**D**) indicate the BgFTZ-F1-F1RE-S1a complex. Arrowheads in (**A** and **B**) indicate supershifted BgFTZ-F1-F1RE-S1a complexes due to the addition of anti-BgFTZ-F1.

To assess whether BgFTZ-F1 could act as an adult-specific factor in the CA, we next analyzed the protein levels of BgFTZ-F1 in CA cells by an electrophoretic mobility shift assay (EMSA) analysis. To this aim, we first searched for a FTZ-F1 response element (F1RE)^38^ in the promoter/enhancer region of the *BgHMG-S1* gene^39^. A detailed inspection revealed the presence of two potential F1REs, F1RE-S1a and F1RE-S1b (Supplementary Fig. S3). EMSA analysis utilizing BgFTZ-F1 protein translated *in vitro* and the F1RE-S1a element as a probe revealed a strong binding complex (Fig. 10A; lanes 2 and 3). In contrast, BgFTZ-F1 was not able to bind to the F1RE-S1b element as revealed by competition analysis (Fig. 10A; lane 4). The identity of the complex was confirmed with an antibody that specifically recognizes BgFTZ-F1^34^ (Fig. 10A; lanes 5 and 6). Once demonstrated that *in vitro*-produced BgFTZ-F1 protein binds to the F1RE-S1a element, we examined the pattern of BgFTZ-F1 protein in CA nuclear extracts during the last two nymphal instars and the adult period. As a previous step, we characterized the occurrence of BgFTZ-F1 protein in nuclear extracts from CA from 5-day-old adult females, by incubating the extract with the F1RE-S1a element (Fig. 10B). We detected a retarded complex whose identity was confirmed with the BgFTZ-F1 antibody (Fig. 10B; lanes 1–3). The identity of the DNA-protein complex was further confirmed as the binding disappeared in CA extracts from *dsBgFTZ-F1*-treated adults (Fig. 10B; lanes 4 and 5). Then, we tested the binding activity of BgFTZ-F1 in nuclear extracts from CA from staged penultimate and last nymphal instars and adult animals. Remarkably, BgFTZ-F1 binding activity was only detected in the imaginal transition and during the entire adult stage (Fig. 10C and D). Taken together, these findings show that BgFTZ-F1 protein competent to bind to the FTZ-F1 response element in the BgHMG-S1 gene is specifically present in the CA cells during the transition from the last nymph to adult and along the adult stage of *B. germanica* females, suggesting that it could act as an adult-specific transcription factor required for JH biosynthesis during this period.

### BgFTZ-F1 is required for JH synthesis in adult *B. germanica*

**Figure 11:**
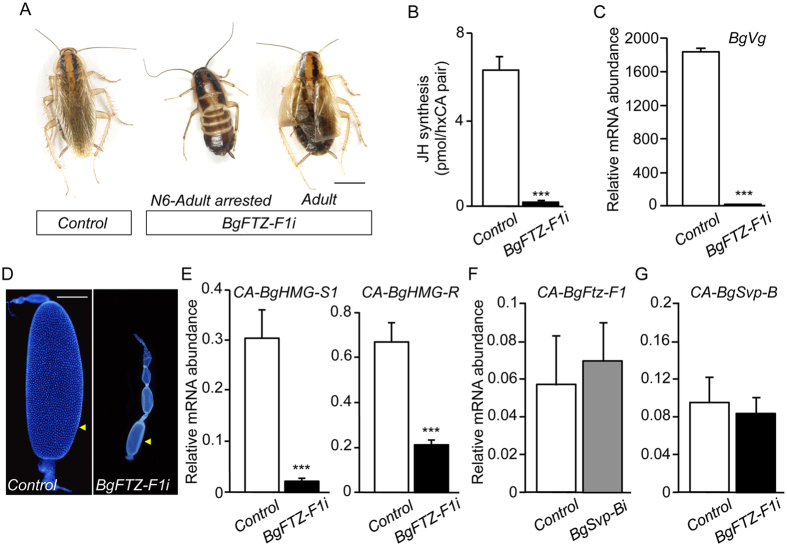
Loss of *BgFTZ-F1* impairs JH synthesis in *B. germanica* adult females. (**A**) Newly emerged sixth instar female nymphs were injected with 3 μg of *dsMock (Control*) or *dsBgFTZ-F1 (BgFTZ-F1i*). Dorsal views of *Control* and *BgFTZ-F1i* animals. *BgFTZ-F1i* animals either arrested development at the transition between the last nymphal instar and the adult stage (left) or molted properly into adults with only minor problems in the extension of the wings (right). (**B**) Rates of JH synthesis by CA incubated *in vitro* from 5-day-old *Control* and *BgFTZ-F1i* adult females. (**C**) *BgVg* mRNA levels in the fat body of 5-day-old *Control* and *BgFTZ-F1i* females, relative to *BgActin5C* mRNA levels, measured by qRT-PCR. (**D**) DAPI-stained ovarioles from 5-day-old *Control* and *BgFTZ-F1i* adult females. Arrowheads indicate basal oocytes. (**E**) *BgHMG-S1* and *BgHMG-R* mRNA levels in the CA of 5-day-old *Control* and *BgFTZ-F1i* females, relative to *BgActin5C* mRNA levels, measured by qRT-PCR. (**F**) *BgFTZ-F1* mRNA levels in the CA of 5-day-old *Control* and *BgSvp-Bi* females, relative to *BgActin5C* mRNA levels, measured by qRT-PCR. (**G**) *BgSvp-B* mRNA levels in the CA of 5-day-old *Control* and *BgFTZ-F1i* females, relative to *BgActin5C* mRNA levels, measured by qRT-PCR. Error bars indicate the SEM (n = 3–10). Asterisks indicate differences statistically significant (***p ≤ 0.0005, Student’s *t*-test). Scale bar in (**D**): 1 mm.

To address the role of BgFTZ-F1 on JH synthesis, we next investigated the effect of *BgFTZ-F1* depletion during the adult stage by injecting *dsBgFTZ-F1* into newly molted last instar nymphs (*BgFTZ-F1i* animals). In agreement with our previous results [36], most of the *BgFTZ-F1i* nymphs (75%; n = 96) arrested development at the imaginal molt (Fig. 11A). However, 25% of *BgFTZ-F1i* nymphs ecdysed properly into adults, with minor problems in the extension of the wings (Fig. 11A), thus allowing the analysis of BgFTZ-F1 functions during the adult stage. Similar to *BgSvp-B*, loss of *BgFTZ-F1* drastically impaired JH production in the CA, *BgVg* expression in the fat body, and egg development (Fig. 11B–D). Likewise, mRNA levels of *BgHMG-S1* and *BgHMG-R* in the CA of *BgFTZ-F1i* adults were significantly lower than those from *Control* animals (Fig. 11E). As a result of these deficiencies, *BgFTZ-F1i* adults did not oviposit nor produced any indication of ootheca formation at the end of the gonadotrophic cycle. Finally, to analyze whether BgSvp-B is involved in the control of *BgFTZ-F1* expression in adult CA cells, we measured *BgFTZ-F1* mRNA levels in the CA of *BgSvp-Bi* adults. As Fig. 11F shows, mRNA levels of *BgFTZ-F1* in the CA of *BgSvp-Bi* adults were not significantly affected, indicating that the role of BgSvp-B in JH biosynthesis is not channeled through the regulation of *BgFTZ-F1* expression in this gland. Similarly, we found that knocking down *BgFTZ-F1* did not affect *BgSvp-B* expression levels in adult CA (Fig. 11G). Overall, our results show that BgFTZ-F1 is a critical factor in the adult-specific control of JH biosynthesis that acts in parallel to BgSvp-B.

## Discussion

During insect development the levels of JH must be appropriately regulated to control the transition between the metamorphic and adult periods. Whereas the metamorphic stage is initiated by the inhibition of the JH biosynthetic activity of the CA cells, the onset of the reproductive adulthood is controlled by the precise re-induction of the CA activity at the imaginal molt^4^,^5^,^6^,^7^,^40^. This critical switch, therefore, constitutes an interesting paradigm for the study of the precise integration of tissue- and stage-specific signals required for the control of a basic developmental process. In the present study, we identify two transcription factors, BgSvp-B and BgFTZ-F1 that are necessary to guarantee the strong activation of key JH biosynthetic enzymes in the CA cells required for the massive production of JH associated to adulthood.

### BgSvp-B is critical for proper CA function

A key aspect of JH synthesis regulation is how the CA-specific expression of JH biosynthetic enzymes is controlled. Our work provides several lines of evidence demonstrating that the nuclear receptor BgSvp-B is a critical transcriptional regulator of JH biosynthetic enzymes in the CA of adult *B. germanica*. To our knowledge, this is the first case in which one isoform of Svp has been linked specifically to the spatial control of JH biosynthesis in insects. To date, Svp had been shown to control the correct development of the photoreceptor cells of the ommatidium^41^,^42^, the formation of the embryonic central and peripheral nervous systems, as well as the fat body, Malpighian tubules and the different cardioblasts in the dorsal vessel of *D. melanogaster*^43^,^44^,^45^,^46^. In the coleopteran *Tribolium castaneum*, TcSvp has been connected with the regulation of the metamorphic process^47^. However, the fact that *Svp* is also highly expressed in the *corpus allatum* of *D. melanogaster* during early embryogenesis^27^, might suggest that this factor could play a conserved role in the transcriptional control of JH synthesis in insects. Consistent with this possibility, it has been recently shown that TcSvp is important for reproduction in adult females of *T. castaneum*, as revealed by the fact that newly eclosed *T. castaneum* female adults treated with *dsTcSvp* show significant egg production impairment and reduced *TcVg* mRNA levels in the fat body^48^.

In addition to the functions described, Svp also exerts regulatory functions in holometabolous insects through the attenuation of the activity of the 20E-signalling cascade by inhibiting the normal functioning of the EcR-USP heterodimer^49^,^50^. In *A. aegypti*, AaSvp interacts with AaUSP at the end of the vitellogenic cycle inhibiting the 20E-dependent transactivation of target genes such as *AaVg*^51^,^52^. Likewise, ectopic overexpression of Svp in larval and pupal stages of *D. melanogaster* leads to lethality at the onset of metamorphosis due to the impairment of the normal activity of the EcR-USP complex^49^. In *B. germanica*, we have shown that the same 20E-triggered hierarchy of nuclear receptors is expressed during nymphal development and controls the metamorphic process, including proper molting^36^. However, in contrast to holometabolous insects, *BgSvp* isoforms seem not to be involved in 20E-dependent processes as revealed by the fact that the *BgSvp*-depleted nymphs developed normally and reached adulthood without any noticeable defect.

It is important to note that, despite *B. germanica* having two isoforms that are highly expressed in the CA cells, the control of JH synthesis is exerted specifically by the *BgSvp-B* isoform. Both isoforms are identical except for the N-terminal end of the molecule. BgSvp-B presents a highly conserved 229 amino acid LBD with a canonical structure formed by 12 α-helices, including the ligand-dependent transactivation domain (AF-2) within α-helix 12, which has been shown to be responsible for the interaction with different coactivator and corepressor proteins^53^. In contrast, BgSvp-A presents a truncated 139 amino acid LBD, with the last 21 amino acids presenting no homology with any other member of the nuclear receptor superfamily. Remarkably, the truncated LBD of BgSvp-A lacks the last 4 α-helices, including the AF-2 domain, which suggests that this region of the protein is key to exert the regulatory functions upon JH synthesis. Further studies are needed to establish the role of the carboxy-end of the LBD in the control of JH biosynthesis by BgSvp-B.

In cockroaches, including *B. germanica*, CA cells proliferate continuously during nymphal development and switch to an adult-specific non-proliferative state at the imaginal molt concomitantly to the increase of JH production (Fig. 7A–D)^24^,^25^. Here, we have shown that the CA cells require the continuous activity of BgSvp-B during nymphal development to maintain their proliferative capacity as revealed by BrdU experiments. Svp has likewise been implicated in the control of cell proliferation in the Malpighian tubules in *D. melanogaster* through the induction of the expression of two cell cycle regulators, the cdc23 phosphatase *string* and *cyclinE*^44^, thus suggesting that the regulation of cell proliferation by Svp is conserved in different insect tissues. Overall, our results show that BgSvp-B is a critical factor that exerts a dual role in the control of the CA in *B. germanica*. First, controlling cell proliferation during the successive nymphal stages, and second, promoting the massive production of JH during the adult period by up-regulating *BgHMG-S1* and *BgHMG-R* expression in the CA cells.

### BgFTZ-F1 is a temporal regulator of JH synthesis

The constant expression of *BgSvp-B* in the CA cells does not correlate with the strong up-regulation of *BgHMG-S1* and *BgHMG-R* and the increase in JH production during the adult period, thus suggesting that other factors must act coordinately to ensure the adult-specific up-regulation of JH biosynthetic enzymes. In this regard, we have identified the nuclear receptor BgFTZ-F1 as responsible for such adult-specificity. Several results support this observation: (1) the binding levels of BgFTZ-F1 in the CA cells correlate well with the expression of *BgHMG-S1* and *BgHMG-R* during the imaginal transition and the adult stage; (2) JH synthesis, *BgVg* induction and oocyte growth are completely impaired in *BgFTZ-F1i* animals; and (3) the mRNA levels of *BgHMG-S1* and *BgHMG-R* are strongly reduced in the CA cells of *BgFTZ-F1i* adults.

It is interesting to note that a strong up-regulation of *BgFTZ-F1* at the imaginal molt is also observed in the prothoracic gland, the tissue responsible of the synthesis of ecdysteroid hormones^34^. In this case, *BgFTZ-F1* induces the degeneration of the prothoracic gland immediately after the imaginal molt, a critical event required for proper adult development in *B. germanica*^54^. BgFTZ-F1 acts, therefore, as a critical adult-specific factor in the two main endocrine glands, the prothoracic gland where induces its degeneration, and the CA where promotes the synthesis of high levels of JH. The role of FTZ-F1 as a stage-specific determinant has been previously demonstrated in the prepupal stage of *D. melanogaster*. In this particular period of development, βFTZ-F1 works as a competence factor for stage-specific responses to the 20E pulse that triggers pupal development^55^. Similarly, in *A. aegypti*, βFTZ-F1 is required for the stage-specific up-regulation of the 20E-dependent genes *E74B, E75A, Vg* and *vitellogenic carboxypeptidase* during the transition to the vitellogenic period^51^,^52^,^56^.

In light of our results, in conclusion, we propose that the high level of JH biosynthesis required during the adult stage of *B. germanica* is achieved by the integration of tissular (BgSvp-B) and temporal (BgFTZ-F1) regulatory inputs. These inputs specifically converge in the CA cells at the imaginal transition and the adult period to guarantee the strong up-regulation of, at least, two important JH biosynthesis enzyme genes, *BgHMG-S1* and *BgHMG-R*. The present work, thus, represents a significant step toward understanding the molecular mechanisms underlying developmental progression in insects by deciphering how the stage- and tissue-specific responses are reached during development.

## Materials and Methods

### Insects

Specimens of *B. germanica* were obtained from a colony reared in the dark at 30 ± 1 °C and 60–70% r.h. All dissection and tissue sampling were carried out in Ringer’s saline using carbon dioxide-anesthetized specimens.

### Cloning of BgSvp cDNAs

Degenerate primers based on the DNA binding domain (DBD) of Svp insect homologs were used to obtain a *B. germanica* homolog cDNA fragment by RT-PCR: forward primer (BgSvp-F1): 5′-AUHGARTGYGTNGTNTGY-3′, and reverse primer (BgSvp-R1): 5′-NCCNVMYCANSRYTANGT-3′. The first amplification was carried out using as a template cDNA generated by reverse transcription from polyA^+^ RNA from 20E-treated UM-BGE-1 cells (derived from early embryos of *B. germanica*), as previously described^32^,^57^. The primers used can be found at S1 Table. The amplified fragment (120 bp) was subcloned into the pSTBlue-1 vector (Novagen) and sequenced. This was followed by 5′ and 3′ RACE (5′- and 3′-RACE System Version 2.0; Invitrogen) to complete the sequence. For 5′-RACE, reverse primer was (BgSvp-R2): 5′-TGAGATTTCTCCTGACACTCCTCT-3′; and for 3′-RACE, forward primer was (BgSvp-F2): 5′-AGAGGAGTGTCAGGAGAAATCTCA-3′. All PCR products were subcloned into the pSTBlue-1 vector (Novagen) and sequenced in both directions. Following this approach, we isolated two different BgSvp isoforms.

### Semiquantitative reverse transcriptase polymerase chain reaction (RT-PCR)

RT-PCR was used to determine the expression pattern of *BgSvp* isoforms. Total RNA was extracted from tissues using the GenElute^TM^ Mammalian Total RNA kit (Sigma). cDNA synthesis was carried out as previously described^18^. Primers for the amplification of *BgSvp* isoforms and the different ecdysone-dependent nuclear receptors can be found at Supplementary Table S1. As a reference the same cDNAs were subjected to RT-PCR with a primer pair specific for *B. germanica Actin5C* as described^57^. cDNA samples were subjected to PCR with a number of cycles within the linear range of amplification for each transcript depending on the tissue and physiological stage.

### Quantitative real-time reverse transcriptase polymerase chain reaction (qRT-PCR)

Total RNA extraction and cDNA synthesis was carried out as described above. Relative transcripts levels were determined by quantitative real-time PCR (qPCR), using iQ SYBR Green supermix (Bio-Rad), in a 20 μl final volume (see Supplementary Table S2 for primer sequences). The qPCR experiments were conducted with the same quantity of organ equivalent input for all treatments and each sample was run in duplicate using 2 μl of cDNA per reaction. All the samples were analyzed using an iCycler and iQ Real Time PCR Detection System (Bio-Rad). For each standard curve, one reference DNA sample was diluted serially.

### RNA interference *in vivo*

RNAi *in vivo* in nymphs of *B. germanica* was performed as previously described^32^,^37^. The primers used to generate templates via PCR for transcription of the dsRNAs are described in Supplementary Table S3. A volume of 1 μl of each dsRNA solution (3 μg/μl) was injected into the abdomen of newly emerged female nymphs. To analyze the effect of the interference in the second adult gonadotrophic cycle, dsRNAs were injected into females in the first day of ootheca transport right after the end of the first gonadotrophic cycle. Eleven days after the injection, oothecas were removed to trigger the onset of the second gonadotrophic cycle.

### Incubation of CA and quantification of juvenile hormone synthesis

JH III biosynthesis by CA incubated *in vitro* was quantified using the methodology previously reported^30^. Basically, individual corpora cardiaca-CA complexes were incubated for 3 hours in 100 μl of 199 medium (Sigma) containing L-methionine (0.1 mM), Hank’s salts, HEPES (20 mM) plus Ficoll (20 mg/ml), to which L-[^3^H-methyl] methionine (Perkin Elmer) had been added to achieve a final specific activity of 7.4 Gbq/mmol. After the incubation period JH III in the medium plus homogenized glands was extracted and quantified.

### Treatments with methoprene *in vivo*

Newly ecdysed adults were topically treated with 1 μg methoprene (isopropyl(E,E)-(RS)-11-methoxy-3,7,11-trimethyldodeca-2,4-dienoate) per specimen in 1 μl of acetone. Controls received the same volume of solvent.

### Microscopy, histological analysis and protein electrophoresis

After dissection, ovaries were fixed in 4% paraformaldehyde and permeabilised in PBS-0,2% Tween (PBT), then incubated for 10 min in 1 μg/ml DAPI in PBT. After two washes with PBT, the tissues were mounted in Mowiol 4–88 (Calbiochem). All samples were examined with a Zeiss Axiophot microscope, and images were subsequently processed using Adobe photoshop.

Proliferation of CA cells was monitored by *in vivo* labeling with 5′-bromo-2-deoxyuridine (BrdU). Nymph and adult insects were injected with a BrdU solution and 24 h later, CA were dissected and fixed in Carnoy’s fixative for 30 min, washed in PBS and incubated in 70% methanol (MeOH) for 10 min, MeOH + 30% hydrogen peroxide (H_2_O_2_) (1:1) for 45 min and 70% MeOH for 10 min. CA were washed in PBS, and then incubated 1 h in PBST-BSA. They were incubated with 2 N HCl for 30 min to denature the DNA and allow access to the anti-BrdU antibody. The tissue was then washed 3 × 10 min in PBS and 2 × 10 min PBST-BSA and placed in PBST-BSA-NGS blocking solution for 30 min. Mouse anti-BrdU (Hybridoma bank) was added at a 1:1000 concentration to a PBST-BSA-NGS solution overnight at 4 °C. Next, the tissues were washed 3 × 10 min PBST-BSA and incubated 30 min in PBST-BSA-NSG prior to 2 h incubation with a peroxidase-labelled anti-mouse IgG secondary antibody (1:100 in PBS-BSA-NGS). Finally, the tissues were washed 3 × 20 min PBS and immunoreactive cells were visualized by incubation in a solution of 3,3′-diaminobenezidine in PBS containing H_2_O_2_ and nickel chloride. Finally, CA cells were examined with Zeiss Axiophot microscope. SDS-PAGE of hemolymph and ovarian proteins was carried out as previously reported^21^.

### *In vitro* transcription/translation

The *BgFTZ-F1* cDNA was cloned into pSTBlue-1 (Novagen)^34^, and transcribed and translated using the TNT coupled reticulocyte lysate system (Promega), according to the manufacturer’s instructions.

### CA Nuclear extracts and EMSA

Preparations of nuclear extracts from *B. germanica* CA were carried out according to the method described^58^. Twenty CA were used for each time-point extraction. Binding reactions were carried out in a 20 μl volume containing 20 CA equivalent of nuclear extracts or 1 μl of the TNT sample, 10 mM Tris-HCl (pH 7.5), 50 mM NaCl, 1 mMMgCl2, 0.5 mM DTT, 0.5 mM EDTA, 4% (v/v) glycerol, 1 μg poly(dI.dC), 1 μg of a single-stranded DNA (5′-TAATACGACTCACTATA-3′), and the indicated amount of competitor DNA or antibody when appropriate. After 15 min incubating at 4 °C, 0.05 pmol of ^32^P-labeled DNA probe was added, and the incubation was continued for another 45 min at the same temperature. The reaction was resolved on 5% nondenaturing polyacrilamide gel run at 4 °C and at a constant voltage of 150 V in 0.5 X TBE. The gel was then dried and autoradiographed. Oligonucleotides (only sense strands are shown) used to generate DNA probes for EMSA were: F1RE-S1a: 5′-GTTCAATTTGTTGACCGAAGGCCGCTATGTTTTCATCC-3′; F1RE-S1b: 5′-GAGTAATAGCCCTAGCCTTAAATTAACATGGGGCC-3′.

## Additional Information

**How to cite this article:** Borras-Castells, F. *et al*. Juvenile hormone biosynthesis in adult *Blattella germanica* requires nuclear receptors Seven-up and FTZ-F1. *Sci. Rep.* **7**, 40234; doi: 10.1038/srep40234 (2017).

**Publisher's note:** Springer Nature remains neutral with regard to jurisdictional claims in published maps and institutional affiliations.

## Supplementary Material

Supplemental Information
