# Supplementary material for: Juvenile hormone biosynthesis in adult Blattella germanica requires nuclear receptors Seven-up and FTZ-F1
Source: Sci Rep. 2017 Jan 11;7:40234. doi: 10.1038/srep40234 (PMC5225475; doi:10.1038/srep40234)
Supplement: Supplemental Information [file srep40234-s1.pdf]

SUPPLEMENTARY INFORMATION

Juvenile hormone biosynthesis in adult *Blattella germanica* requires nuclear receptors Seven-up and FTZ-F1

Ferran Borras-Castells, Claudia Nieva, José L. Maestro, Oscar Maestro, Xavier Belles and David Martín*

Institute of Evolutionary Biology (CSIC-Universitat Pompeu Fabra)
Passeig Marítim de la Barceloneta 37-49, 08003 Barcelona, Spain.

*Corresponding author: Telephone number: 34 932309640; Fax number: 34 932211011; E-mail address: david.martin@ibe.upf-csic.es

Supplementary Information

Supplemental Table 1. Primers used to amplify the different target genes for semiquantitative RT-PCR analysis. The amplicon size is indicated. F: forward primer; R: reverse primer.

Target	Fragment	Primers sequence
BgSvpA	509 bp	F: 5'-GTCGAGGTATGGACAATGCATGCA-3'
		R: 5'-TGCGTTAGAGAACAAGTAGCTCCAGT-3'
BgSvpB	526 bp	F: 5'-GTCGAGGTATGGACAATGCATGCA-3'
		R: 5'-AGATGGTAAACGGAGCAAGAGCTT-3'
BgHMG-S	483 bp	F: 5'-GAAGTCTCTTGCTCGCCTCGTC-3'
		R: 5'-TTGTGTTCCCTCGTCTCCATCGT-3'
BgHMG-R	733 bp	F: 5'-CACTTGCAACAACTGAGGGC-3'
		R: 5'-GAAGGCATGGTGGAGGATAC-3'
BgE75A	324 bp	F: 5'-TTAGTGCTAGTGCAATGTGCTATTGA-3'
		R: 5'-ATGGAGCACTGTTGGTTCTTGGTA-3'
BgHR3-A	631 bp	F: 5'-GGCGCACCAAAAGTGGATTAATTGA-3'
		R: 5'-CGCTGATGTCGTACGGCATCT-3'
BgHR4	477 bp	F: 5'-AACTCTGGAGCTGTTTACAACTTGTA-3'
		R: 5'-CACTTGTGCGTTAATAATCTTGTGT-3'
BgFTZ-F1	601 bp	F: 5'-GAGGAATTCCGTCTTCTTCAAGGA-3'
		R: 5'-GGACTTTGTCAACAATTCATTTCAGT-3'
BgActin5C	308 bp	F: 5'-TCGTTCGTGACATCAAGGAGAAGCT-3'
		R: 5'-TGTCGGCAATTCCAGGGTACATGGT-3'

Supplemental Table 2. Primers used to amplify the different target genes for quantitative RT-PCR analysis. The amplicon size is indicated. F: forward primer; R: reverse primer.

Target	Fragment	Primers sequence
BgSvpA	120 bp	F: 5'-GTAAGTGTGACAAACAGCAGTCT-3'
		R: 5'-TGCGTTAGAGAACAAGTAGCTCCAGT-3'
BgSvpB	110 bp	F: 5'-ACATCGAAAGTCTCCAAGAGAAATC-3'
		R: 5'-AGATGGTAAACGGAGCAAGAGCTT-3'
BgHMG-S	200 bp	F: 5'-CTTCGCTTTACGGAGGTTTGGTC-3'
		R: 5'-GCTGCGGCTTGATGTGCGAGAG-3'
BgHMG-R	200 bp	F: 5'-TTGTAGCTGATGGAATGACTCGT-3'
		R: 5'-AGCAATGAAGCGTATAAACAAATG-3'
BgVg	200 bp	F: 5'- CTACATTGGAAGTGAGGACAGC-3'
		R: 5'-CCAAAGTTTTAGGATCAGTAGGTG-3'
BgFTZ-F1	120 bp	F: 5'-TTCGAACTCTTGCAGAACCAGAC-3'
		R: 5'-CAGTCGACCTGAGAGAACAAATTC-3'
BgActin5C	213 bp	F: 5'-AGCTTCCTGATGGTCAGGTGA-3'
		R: 5'-TGTCGGCAATTCCAGGGTACATGGT-3'

Supplemental Table 3. Primers used to amplify the sequence designed to specifically interfere nuclear receptors BgSvp-A, BgSvp-B and BgFTZ-F1. The fragment length is indicated. F: forward primer; R: reverse primer.

dsRNA	Fragment	Primer sequence
dsBgSvp-1	356 bp	F: 5'-GTCGAGGTATGGACAATGCATGCA-3'
		R: 5'- TATACTCCGCAGAGTCTACGTGAAG -3'
dsBgSvpA	247 bp	F: 5'- GTAAGTGTGACAAACAGCAGTCT-3'
		R: 5'- TAGGAGTTTAAAAGTAAAGTGACA -3'
dsBgSvpB	388 bp	F: 5'- ACATCGAAAGTCTCCAAGAGAAATCA-3'
		R: 5'- AGCATTGATGTGCATACAAGCCTT-3'
dsBgFTZ-F1	502 bp	F: 5'-GAATAGTTCAGGGCTTTTTGAAGCT-3'
		R: 5'-CGACGCATGTGTAGTCCTTCTT-3'

Supplementary Figure Legends

Supplementary Figure 1. Domain comparison of *B. germanica* Svp isoforms with other Svp/COUP-TF nuclear receptors. Letters above BgSvp-A indicate functional domains. Numbers within each domain indicate the number of amino acids. The percentages of identity between corresponding domains of BgSvp-B and the other orthologs are indicated below each domain. Sequences and species considered are BgSvp-A and BgSvp-B from *B. germanica* (this study), *Drosophila melanogaster* (DmSvpB; accession number: NP_524325.1), *Tribolium castaneum* (TcSvpB; EFA11548.1), *Aedes aegypti* (AaSvpB; XP_001655965.1), *Bombyx mori* (BmSvpB; BAB55582.1), *Apis mellifera* (AmSvpB; XP_392402.2), *Acyrthosiphon pisum* (ApSvpB; XP_001944021.1), *Homo sapiens* (HsCOUP-TF1; NP_005645.1, and HsCOUP-TF2; NP_066285.1), *Mus musculus* (MmCOUP-TF1; EDL37125.1; and MmCOUP-TF2; AAH42484.1), *Danio rerio* (DrCOUP-TF; NP_571255.1) and *Xenopus laevis* (XlCOUP-TF; AAD42224.1).

Supplementary Figure 2. Silencing of *BgSvp* by RNAi in vivo in sixth instar female nymphs of *B. germanica*. (A) Scheme of BgSvp-A and BgSvp-B domain organization showing the region used to generate the different dsRNAs. (B) Effectiveness of *BgSvp*-RNAi. A dose of 3 µg of *dsMock* (*Control*) or *dsBgSvp-1* (*BgSvpi*) targeting *BgSvp-A* and *BgSvp-B* isoforms simultaneously were injected into newly emerged N6 nymphs, and mRNA levels of *BgSvp-A* and *BgSvp-B* in the CA were measured 6 days later by qRT-PCR. Error bars represent SEM (n = 10). Asterisk indicates differences statistically significant as follows: *$p \leq 0.0001$ (Student's *t* test).

Supplementary Figure 3. The nucleotide sequence of the promoter/enhancer region of the *BgHMG-S-1* gene. The two putative FTZ-F1 response elements (F1RE-S1a and F1RE-S1b) are underlined.

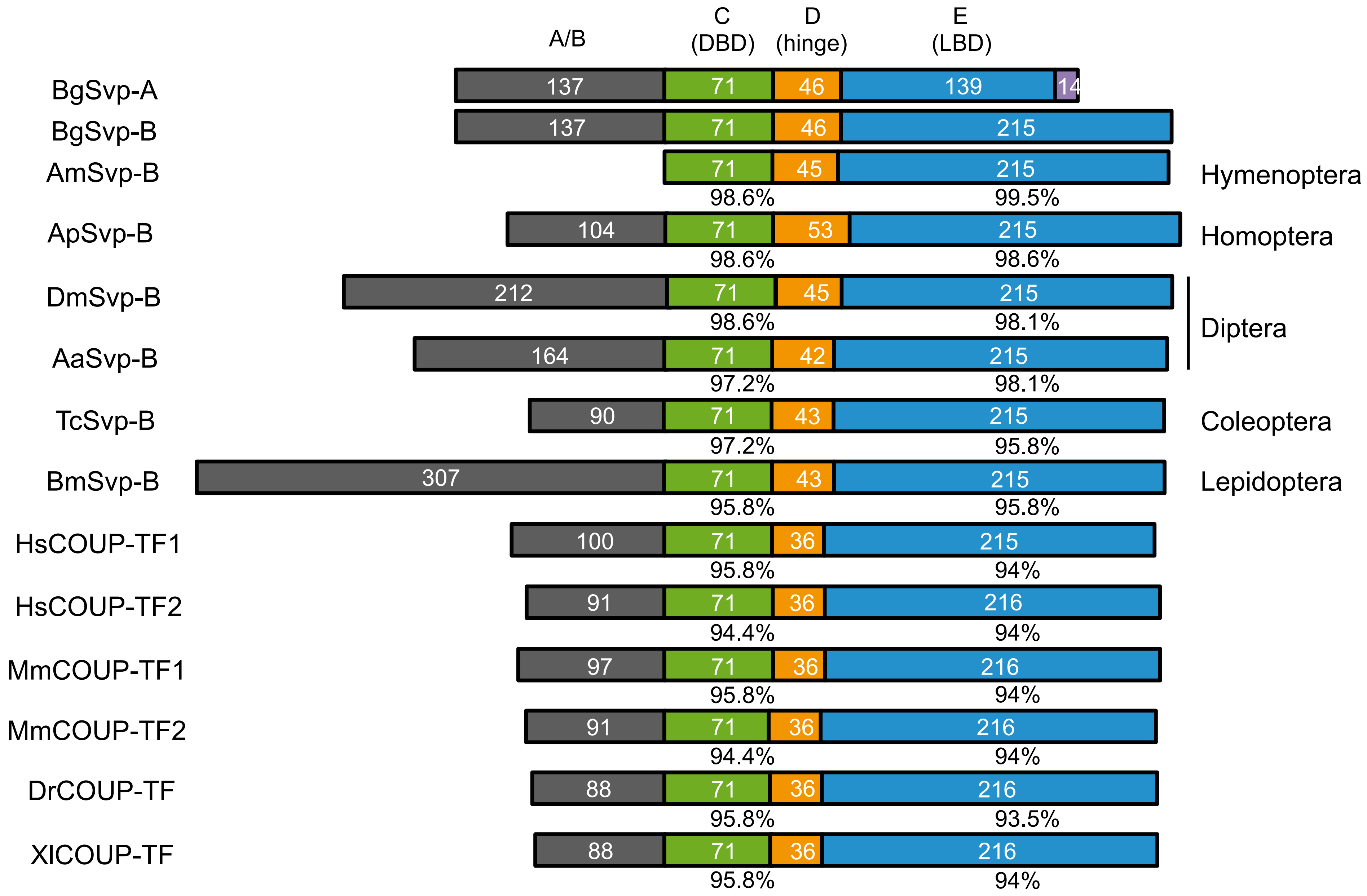

Supplemental Figure 1

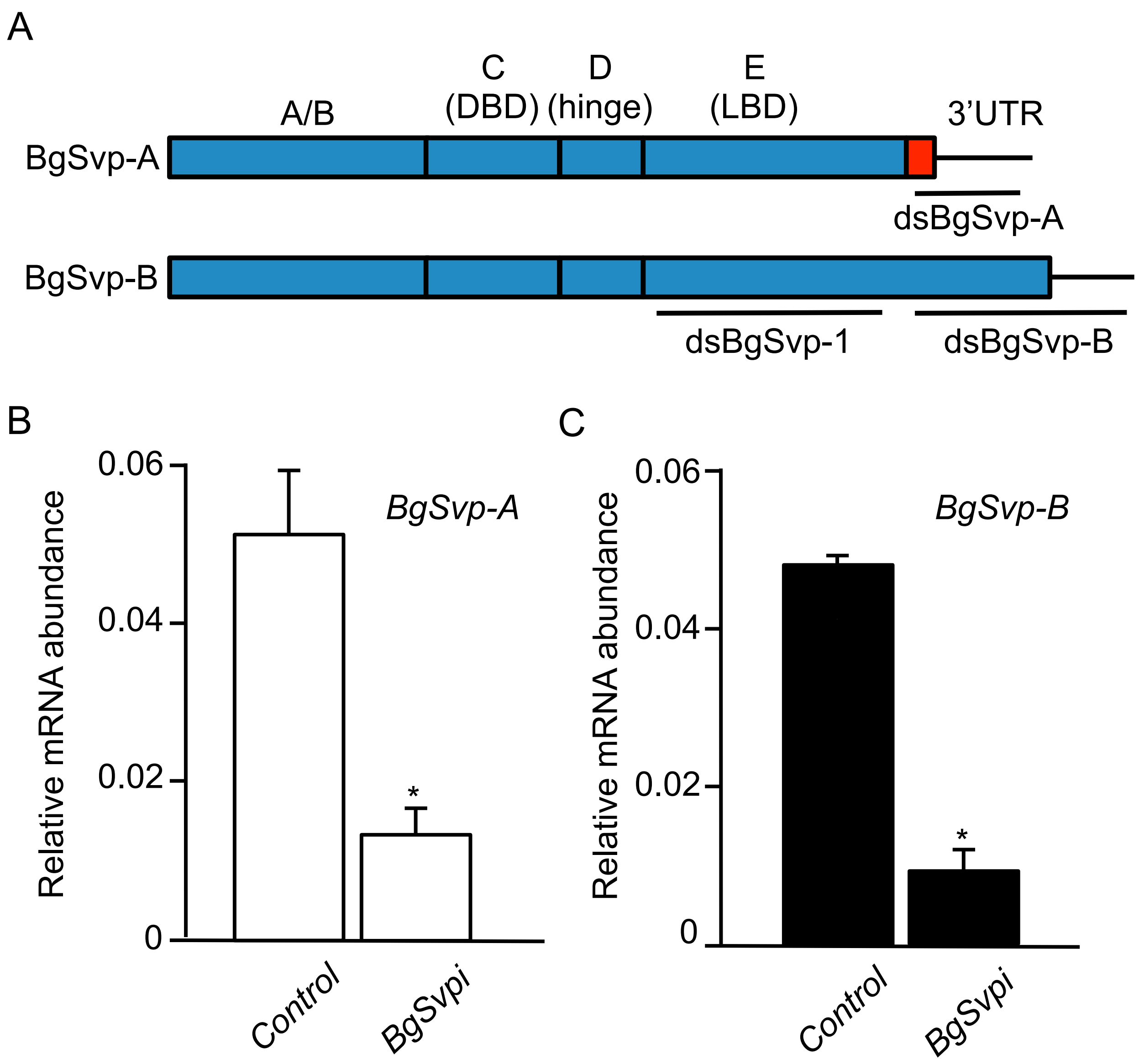

Supplemental Figure 2

```
-827                                GAATTCAGCACTGGTGTGGAGTGAAGAATCATATTCCTACCGACCAT
-780  TGGCTCCTGAGTAAAATGGACTTTCTTTTAGTTGGCCAGTGTTTGAATTGTTCAGGAAAACTGCA
-715  GACCATGCAATCCAACCATTCCTCACTACCACTCGGTTTTTCAGATGCAAAAACAACAGAGATGA
-650  ATTTGAGTAATAGCCCTAGCCTTAAATTAACATGGGGCCTGCCTATTTATTATTATTATTATTAT
                      F1RE-S1b
-585  TAATAATCTGCTGGTGGGAAGCATGGTCTGCAGTGTACCTGAAGCAGGATTCACACCAGTATTTT
-520  TTTTTTAATTATTGGGATATATATTAGTTCTATGTAATGCAGCATTAGAAACATTATTTCATGGA
-455  CATGAAGCCAGGATCTCATACAGAAAGTCTATATCTAAACTTTCACATGACTGTAACAAGTGCCG
-390  TTAATCATTTAAAAAAAAGCTAAATGTAGGGCAAGTTTACTAATCAAGTTTGCAGAAATTATTAG
-325  CAGCTGCTTTTTTCAACAGTGGGAATAGTTTAACCTAAGACAACACTTGTGAAATTGAGGTTATG
-260  TCGTATTCACCTGTGCTTCATATTGTTCAATTTGTTGACCGAAGGCCGCTATGTTTTCATCCATT
                                               F1RE-S1a
-195  ATTATTTATTCAAAACAAAATTAAAGCTCTCGGGTTTAGCTACAAAGAACTAAATCCTAACATCC
-130  GTGAGTGACAAATTACCCTAAAACTCGTTATTCAAACAACGACTGTCGCCATTGTGATCCTGGGG
 -65  ACGTATGGAACAGAAGAAAGTCGAAGGAATGGCAGGAATGCGGCCGCTATCATTCAACTATTTTG
  +1  TAGATCGTGTTTCGAGGTTAGGATGTCACGCAAAGAGGGCATGACTCAAAGCTAGCAGTCTGTGC
 +66  TCAGTCTGTCCTAAGACGATG
```

Supplemental Figure 3
